# Fifth Annual Meeting of the Mississippi Valley Association of Dent. Surgeons

**Published:** 1849-10

**Authors:** 


### FIFTH ANNUAL MEETING OF THE MISSISSIPPI VALLEY ASSOCIATION OF DENT. SURGEONS.

The Society met pursuant to adjournment, at Louisville, September 11th, 1849, the meeting being held in the Temperance Hall.

At 10 o'clock, A. M., the President, Dr. Goddard, took the chair, and called the meeting to order, when the following members were present: Drs. Goddard, Jas. Taylor, Leslie, Allen, Taft, Curtiss, Vaneman, Bonsall, Griffith, and McCullom.

The president's address being called for, he arose and read a paper of great interest.

On motion of Dr. Taylor, said address was accepted, and ordered to be published.

Dr. J. W. Bright, of Louisville, was presented by Dr. Taylor as a candidate for honorary membership in the society, who also moved his admission, which passed unanimously.

Dr. Bright being present, he was introduced to the society, when in a few brief remarks he thanked it for the honor conferred, approved highly of the objects of the society, and gave many cogent reasons to prove the necessity of such organizations.

On motion of Dr. Griffith, the chair appointed Drs. Taylor, Leslie, and Curtiss a committee to examine the specimens of mineral teeth, presented in competition for the society's premiums offered at the fourth annual meeting.

The corresponding secretary (Dr. E. Taylor,) being prevented from attending by indisposition, sent in the following report by the hands of Dr. Jas. Taylor:

### REPORT OF THE CORRESPONDING SECRETARY.

The Corresponding Secretary reports that he notified the delinquent members of the Society, as instructed by the Society, but that he was not favored with a response in any case, and that their names were, in consequence, omitted in the published list.

Respectfully submitted,

E. TAYLOR, C. S.

CORRESPONDING SECRETARY'S REPORT ON TEETH FOR PREMIUMS.

The Corresponding Secretary reports that he gave notice as instructed by the Society, to all the manufacturers of mineral teeth that he could hear of, of the premium offered by the Society, and respectfully submits the lots received for examination.

E. TAYLOR, C. S.

The Publishing Committee being called upon, reported the following:

The Publishing Committee, in submitting to the Society a record of our acts since the last meeting, would report that the Register has been regularly published, and that the last number of the second volume, although somewhat delayed in consequence of the prevailing epidemic, yet it has been sometime since distributed to the subscribers.

The committee would draw the attention of the Society to the improved appearance of the Register, and offer this as an excuse for the increased cost of publishing. It was found utterly impracticable to issue the Register according to the design of the Society, and make it a creditable periodical, without incurring some additional expense. The committee regret that they have not yet the means at command to make it such a work as they desire. The work could be much embellished, and made more attractive, if such cuts and illustrations as at times are necessary to properly illustrate certain subjects of a mechanical nature, were introduced. The committee would also draw the attention of the Society to the importance of having regular contributions from the different members, so that at no time there shall be a deficiency of proper matter to make up a number in proper time for the publisher. The neglect in this particular has caused the committee much additional labor, and that frequently at such times as made it particularly harrassing.

The committee would also suggest the importance of increased exertion on the part of the members of the Society in procuring subscribers. There are many practicing Dentists in the west that take no periodical at all; and if as is supposed by those who are careful observers, that periodical literature is always several years in advance of science, as received by regular book publication, there must be many in the profession whose interests would be advanced, and the profession elevated, if they could be induced to avail themselves of the earliest information given.

| | |
|---|---|
| The additional amount received for subscriptions and advertisements since the last report, | $136.00 |
| The amount then on hand, | 96.66 |
| | $232.66 |
| The amount expended for 2d Volume of Register and stationery, | 217.90 |
| | 14.76 |
| The committee have drawn from the treasury of the Society, during the last year, | 60.00 |
| Leaving in the hands of your committee, | $74.76 |

J. TAYLOR,

W. H. GODDARD, } *Committee.*

On motion, it was

*Resolved*, That eight dollars additional be appropriated to meet the contingent expenses of the Register for the past year.

On motion of Dr. Bonsall,

*Resolved*, That the chair appoint a committee of three to consider the project of altering or amending the present mode of publishing the Register:

Whereupon, the President appointed Drs. Taylor, Griffith, and Allen said committee.

The Treasurer presented his annual report, which was read and referred to an auditing committee, consisting of Drs. Griffith, Vaneman, and Curtiss.

TO THE MISSISSIPPI VALLEY ASSOCIATION OF DENTAL SURGEONS.

Having made arrangements to retire from the arduous duties of the profession, I hereby resign my membership in the Mississippi Valley Association of Dental Surgeons. In taking formal leave of the members of the Association, with whom I have had the pleasure of acting in the noble efforts they are making to elevate the profession, to which the energies of nearly twenty years of the *best* period of my life have been devoted, I cannot but make grateful acknowledgments for the kindness and courtesy that has at all times been extended to me by all the members of the Association, and by all the members of the profession with whom I have had intercourse. With a profession that has received so large a part of the *prime* of my life, and with which are blended many of my most pleasant memories, I expect not, nor desire any severance of feeling; and although I may never be called upon to assume its arduous labors or anxious responsibilities, yet I shall never cease to bear it a lingering attachment, and cherish a most lively interest in its well being. Respectfully yours,

E. TAYLOR.

On motion, the Society accepted of the resignation of Dr. E. Taylor, but expressed its deep regret at the loss of so active a member.

Dr. Leslie moving, Dr. Griffith seconding,

Dr. E. Taylor was unanimously elected an honorary member of the Society.

On motion, the Society adjourned to meet at 3 P. M.

AFTERNOON SESSION.

Society called to order by the president, who announced an address by Dr. Leslie, to be now in order.

Dr. L. occupied the attention of the Society for sometime, in reading a paper on Metallurgy, which, on motion, was accepted, and ordered to be published.

The auditing committee presented the following report:

TO THE MISSISSIPPI VALLEY ASSOCIATION OF DENTAL SURGEONS.

Your committee appointed to examine the Treasurer's books and vouchers, beg leave to report that they have found his account correct, and that there is now left in his hands, a balance of $69.60.

S. GRIFFITH,
G. L. VANEMAN,
J. G. CURTISS.

Dr. Griffith being called upon for his address, arose and delivered one on Maxillary Abscess, which, after a short and interesting discussion, was, on motion, accepted.

Dr. Taft's address on extraction of teeth being next in order, the Doctor read a paper which produced an animated discussion, after which, it was, on motion, accepted.

On motion of Dr. Taylor, the following resolution was adopted:

*Resolved,* That this Society view with pleasure the success which has attended the recent plastic operation of our professional brother, Dr. S. P. Hullihen, of Wheeling, Va., on Miss M——, and that the skill exhibited by the Doctor in that delicate and important operation, is worthy of our highest consideration and imitation.

On motion, the Society adjourned to meet in the office of Dr. Goddard, at 7½ o'clock.

EVENING SESSION.

Society met pursuant to adjournment. The address of Dr. Curtiss being next in order, the Doctor read a paper on the use of the file upon the teeth, which, after some discussion, was, upon motion, accepted.

Dr. Taylor, from the examining committee, presented the name of Dr. S. C. Gray, of Cincinnati, as an applicant for membership in the Society, whereupon the Society went into an election by ballot, when the president announced him duly elected.

The remainder of the evening was spent by the Society in an interlocutory, embracing several principles and modes of practice, which proved so advantageous as to induce the Society to resolve they would have a repetition of the same at their next meeting.

On motion, Society adjourned to meet at 9 o'clock, A. M., at the Hall of the Sons of Temperance.

SECOND DAY, 9 o'clock, A. M.

Society met according to adjournment; meeting called to order by the president.

Minutes of first day read and approved.

The president announced the order of the day to be the reading and consideration of the report of the committee appointed at the fourth annual meeting of the Society, to report on the best mode of filling teeth.

Dr. Jas. Taylor, from that committee, stated, that owing to the absence of two of its members, the committee had not been able to confer on the subject; but he had received from each of the absentees a communication, exhibiting their views, which he would read to the Society, in order to elicit discussion on this important point of practice.

After reading and discussion, these papers were, upon motion, referred to the publishing committee, with instructions to publish an abstract of the same.

The committee to whom was referred the subject of altering or amending the present mode of publishing the Register, reported by recommending the continuance of its publication as at present conducted.

On motion,

*Resolved*, That the Society do now go into an election of officers to serve for the coming year.

After balloting, the following gentlemen were declared duly elected:

JAS. TAYLOR, President.
JOHN ALLEN, 1st Vice President.
SAMUEL GRIFFITH, 2d do.
A. M LESLIE, 3d do.
W. H. GODDARD, Corresponding Secretary.
JOHN ALLEN, Recording Secretary.

J. Taft,<br>B. T. Whitney,<br>A. Berry, } Examining Committee.

S. P. Hullihen,<br>G. L. Vaneman,<br>J. G. Curtiss, } Exec. Com.

Jas. Taylor,<br>W. H. Goddard,<br>A. M. Leslie, } Pub. Com.

On motion of Dr. Goddard, it was unanimously

*Resolved,* That this Society pledge itself to the Publishing Committee, to relieve it from any personal liability which its members may necessarily incur in the publication of the Register.

On motion, adjourned to meet at 3 o'clock, P. M.

AFTERNOON SESSION, 3 P. M.

Society met according to adjournment.

The committee on mineral teeth being called upon for their report, presented the following:

Your committee beg leave to report, that in examining the various specimens of teeth placed before them, and recommending such as they consider entitled to the premiums of the Society, they have sought to be guided solely by the resolution by which the premiums were offered, and the characteristics of the teeth.* In no case was the committee aware of the name of either of the manufacturers. *All* specimens were subjected to the *same* tests, and compared in the same particulars. Those specimens which the committee recommend to the Society as best entitled to the premiums, they have marked A and B. In both of them they find pivot, plate, and gum teeth, molars and bicuspides. The plate and pivot teeth in both are nearly of an equal grade of manufacture. The gum teeth, in parcel A, in form and color

---

*Extract from minutes of the fourth annual meeting:

"On motion of Dr. E. Taylor, the following resolution was passed:

*Resolved,* That the Society offer to the manufacturer, a gold medal worth twenty dollars, for *one hundred of the best teeth* offered; including specimens of gum teeth, of molars and bicuspides, and common plate and pivot teeth, in equal proportions; and that the second best, of the same amount and character, be presented a silver medal worth ten dollars."

are, in your committee's opinion, preferable to those in B. The committee would desire to call the particular attention of the Society to a few teeth of a special class, found in parcel B, which the maker styles "Dens Perfectiones." They believe they have never before seen artificial teeth in which the materials were so happily blended, as so successfully to imitate that yellow class of teeth possessed in general by those enjoying a sound, vigorous constitution. Their excellent imitation of the natural teeth in form, is also worthy of all praise—still your committee cannot think them likely, on this account, to supercede those now in use—but would add that, comparing them with an *equal number* of any lot before them, they command their decided preference.

Your committee however, being guided (and the Society pledged,) by the resolution quoted at the opening of this report, they feel bound to recommend that the Society award to the maker of specimen marked A, the gold medal, believing it to contain the best *hundred* teeth, and to the maker of specimen marked B, the silver medal, judging it to contain the second best *hundred*. All of which is respectfully presented.

JAS. TAYLOR,<br>A. M. LESLIE,<br>J. G. CURTISS, } Committee.

On motion, this report was accepted, and the committee discharged.

The Society, after due inspection of the specimens, adopted the report, and awarded the premiums as recommended by the committee.

The president then announced Messrs. Jones, White and McCurdy, to be the manufacturers of the specimen marked A, and Mr. James Alcock, manufacturer of that marked B.

On motion of Dr. Taylor,

*Resolved*, That twelve of the "Dens Perfectiones" be deposited in the museum of the Ohio College of Dental Surgeons, for preservation and future reference.

On motion of Dr. Taylor, it was made the duty of the Treasurer to have prepared the gold and silver medals, with suitable device and inscriptions, expressive of the Society's object in awarding the same, and to have the medals presented to the successful competitors.

By resolution, it was made the duty of the Corresponding Secretary, to make known to Messrs. Jones, White and Mc Curdy, and Mr. James Alcock, the award just made by the Society.

Dr. Taft offered the following resolution, and moved its adoption, which carried unanimously.

*Resolved*, That the thanks of the Society be awarded to the Editors of the Register for their untiring energy in conducting its publication.

On motion of Dr. Leslie,

*Resolved*, That when we adjourn, we do so to meet the second Tuesday in September, 1850, at the lecture room of the Ohio College of Dental Surgery.

On motion of Dr. Goddard, two dollars were appropriated to pay the person in attendance at the Hall. Also

*Resolved*, That the thanks of the Association be presented to the Trustees of the Sons of Temperance Hall, for the use of their rooms.

The following gentlemen were appointed essayists for the year 1850:

Dr. Whitney, on Professional Etiquette;
Dr. H. McCullom, on Filling Teeth;
Dr. Bonsall, on Alveolar Abscess;
Dr. John Allen, on Artificial Gums;
Dr. Vaneman, on Blow-pipe;
Dr. Taft, on Dentition;
Dr. Gray, Connection of Chemistry and Dentistry.

On motion of Dr. Gray,

*Resolved*, That the thanks of this Society be tendered to Drs. Goddard and Griffith, for their generous hospitalities extended to the members during its present session.

On motion, adjourned.

W. H. GODDARD, Pres't.

John Allen, Recording Sec'y.

